# Fractional Calculus and the Future of Science

**DOI:** 10.3390/e23121566

**Published:** 2021-11-25

**Authors:** Bruce J. West

**Affiliations:** Center for Nonlinear Science, University of North Texas, Denton, TX 76203, USA; brucejwest213@gmail.com

The invitation to contribute to this anthology of articles on the fractional calculus (FC) encouraged submissions in which the authors look behind the mathematics and examine what must be true about the phenomenon to justify the replacement of an integer-order derivative with a non-integer-order (fractional) derivative (FD) before discussing ways to solve the new equations. The desired articles are intended to provide the reader with a window into the future of specific science disciplines by peering through the lens of the fractional calculus (FC) and suggesting how what is seen entails a difference in thinking about that area of science. Thus, a perfect submission would be more about the implications and utility of the FC than about its formal structure in chemistry, epidemiology, sociology, psychology, physics, or any of the other scientific disciplines. Imaginative articles that implement FC in new and interesting ways that reveal its transformational nature, including, but not limited to, such things as: how a fractional derivative in time incorporates memory into the solution of the dynamic description of an earthquake, a brain quake, or a crash in the stock market; how the fractional derivative in space incorporates spatial non-locality into the solution of the complex dynamical descriptions of a riot, the collective intelligence of social groups, or the neuronal activity of the brain. Finally, we are interested in how the combined fractional derivatives in time and space of functional measures of uncertainty incorporate both memory and nonlocality into the phase space solution to capture the limited uncertainty of an ensemble of fractal trajectories, or the scaling behavior of complex dynamical networks.

As West points out [1], Sir Isaac Newton transformed *Natural Philosophy* into today’s *Science* by focusing on the fundamental nature of motion, and he did so by using geometry in a way that resonated with the scientific community of his day. What Newton accomplished was to reveal what was entailed by fluxions (the differential calculus) without explicitly referencing them and in so doing he convinced generations of scientists of the value of their analyzing how physical phenomena change in space and over time. Whether by conscious plan or by serendipity, Newton cleared a path for less talented investigators to follow and contribute to the nascent discipline of mechanics and thereby determined the direction of quantitative reasoning in physics for the next three centuries.

This was the starting point for a discussion of how science, through its intermittent turning of its investigative tools on itself, has reached another epoch of transition. However, unlike the paradigm shifts within a scientific discipline introduced by Thomas Kuhn in 1962 the present shift addresses the whole of science. West argues that the dominance of Newton’s world view is drawing to a close and he weaves the threads of chaos theory, fractals, non-ergodic behavior of dynamic systems and fractional kinetic theory into a fractal tapestry of the physical, social, and life sciences. It is the complexity of this tapestry that is shown to be fundamentally incompatible with Newton’s notions of space and time.

Angstmann and Henry (AH) [2] address the discipline of chemistry, specifically the equations governing the time evolution of population densities of chemical species taking into account the spatial movement and their interactions using reaction–diffusion equations. They discuss the failure of classical techniques to properly describe diffusion and review fractional subdiffusion resulting from particles being trapped for arbitrarily long times, which are modeled using the continuous time random walk (CTRW) model. This fractional subdiffusion is characterized by the mean square displacement of a chemical population spreading as a sublinear power law in time and is described using a Caputo fractional derivative in time in a reactive–subdiffusion equation.

AH emphasize that the main lessons learned from their analysis: (i) The governing equations are different depending on whether newborn particles inherit the waiting times of their parents. (ii) Birth and death terms must be treated differently. (iii) In the case where particles are removed, but not instantaneously at the start of the waiting times between jumps, the reaction and subdiffusive terms are not additive. They go on to explore the analytic solution to several exemplars, including a mass-conserving tempered time fractional diffusion equation that is subdiffusive for short times but manifests standard diffusion at long times.

Machado [3] chose to explore the fractal nature of financial time series using the Dow Jones industrial average (DJIA) but avoided using standard time series analysis. Instead, he uses multidimensional scaling (MDS) together with the concepts of distance, entropy, fractal dimension, and the FC. Introducing ten distinct definitions of distance, most of which I had not previously encountered, he was able to generalize MDS as an extension of the traditional metric formulation to construct a smooth non-Euclidean space. In this space, the fractal dimension and entropy measures for analyzing the three-dimensional portraits produced by the generalized MDS are interpreted.

Several known relations between the fractal dimension, using the box counting definition, and the idea that a random variable’s entropy is its average level of “information” of the corresponding PDF, are used to define the information as an entropy of non-integer order α. This parameter α gives an extra degree of freedom to adapt the sensitivity of the entropy calculation to each specific dataset. Machado points out that time is viewed as a continuous and linear flow so that any perturbation is automatically assigned to the variable under analysis. Stated differently, since people are entities immersed in the time flow, apparently, we are incapable of distinguishing between perturbations in the time or the measured variable. Machado’s analysis explores an alternative strategy to that of time series analysis for reading the relationship between the variables.

In the reaction–diffusion equation discussed by AH the chemical reaction term was modeled using the Verhulst equation for population growth. In 1798, Malthus argued that the integer-order rate equation produced an exponential population growth and 40 years later Verhulst replaced the constant growth rate of Malthus with one which decreased linearly with the growing population to mitigate the dire predictions of Malthus. The Verhulst (logistic) equation has the advantage of being one of the few nonlinear equations that has an analytic solution depicting a sigmoidal growth to a finite maximum population. Izadi and Srivastava (IS) [4] recount this bit of history and note that the integer-order derivative has been replaced by a fractional derivative in applications in numerous disciplines of science and engineering. They go on to point out that for most fractional differential equations (FDEs) there is to date no possibility of finding an exact analytic solution.

IS provide a brief review of the analytic and numerical methods that have been developed and applied for the FDEs which are based upon various loosely related models of real-world problems. Given the popularity of the fractional logistic equation (FLE) in the modeling of such phenomena as the growth of tumors in medicine, IS use it as a prototype on which to utilize the local discontinuous Galerkin (LDG) discretization approach for numerically solving the FLE. Given that the FLE has a second-order non-linearity IS rewrite it as two linear first order FDEs to apply the LDG scheme. Consequently, LDG is employed to discretize the resulting system, as well as the fractional operator. The mathematical details of the technique are presented along with comparison with alternative numerical and approximation approaches.

The transport of particles through continuous media is described by transport equations often based on general principles, such as conservation of energy and momentum. As pointed out by Masoliver [5], in general these transport equations are unsolvable nonlinear integrodifferential equations. He elected to discuss transport processes using the telegrapher’s equation (TE) and its generalization to the fractional case, emphasizing that random walk (RW) models are fundamental in describing TEs because they try to reproduce the microscopic mechanisms of transport. In this way he accounts for “diffusion with finite velocity”.

The integer-order TE (IOTE) at early times behaves like a wave front and at late times like ordinary diffusion, it is generalized to a fractional-order TE (FOTE) in transport through highly disordered media, for instance, random media or fractal structures. Masoliver shows how this is done using fractional RWs resulting in a three-dimensional FOTE that is fractional is both space and time. The two different dynamics governing transport are fractional wave behavior at early times and fractional diffusion behavior as late times. Masoliver also shows analytic solutions for various combinations of IO-time, FO-space, FO-time and IO-space derivatives in one, two and three dimensions.

There are some investigators whose papers I always anticipate reading because I know that in addition to my gaining technical knowledge the author will put their work into a context that I would have missed left to my own resources. Professor Mainardi [6] falls into that category and his review paper falls into another category as well, that being: “Everything you wanted to know about _?_ but were afraid to ask.”, here the blank is filled in with the Mittag–Leffler function (MLF). Just as the exponential function is the workhorse of linear IO differential equations, the MLF is the workhorse of linear FO differential equations, and the latter reduces to the former when the FO index goes to unity. His paper is written with the skill and insight that only one who has worked with the leaders in the field and has himself made lasting contributions to our understanding could manage.

His survey interleaves and draws connections among stochastic processes, such as the fractional Poisson process, the thinning of renewal processes, using the MLF. The CTRW is used to generalize the classical Kolmogorov–Feller equation (KFE) to the fractional KFE (FKFE) where the MLF is the waiting-time PDF. The fractional diffusion-wave equation has solutions to boundary value problems in terms of Wright functions that are inverse Laplace transforms of two parameter MLFs and are Greens functions in the solutions to specific boundary value problems.

Big Data (BD) and Machine Learning (ML) are two of the more visible areas of research in which investigators are working to span the gap separating the understanding based on modeling in social and life sciences from the more quantitative models of physics and engineering. Niu et al. (NCW) [7] maintain that the future success of these research activities is tied to the successful application of the FC and fractional order thinking (FOT) to the understanding of complex systems, to improving the processing and control of those systems and even to extending the enabling of creativity itself. The heart of the matter is that BD and ML seek to characterize complexity and of the ten characteristics used to describe BD *variability* is selected by NCW as the most important.

The complexity observed in most BD is almost invariably manifest through inverse power law (IPL) resulting from the processed data. The heavy-tailed nature of multiple PDFs is discussed along with its connection to the FC through fractional diffusion equations that are fractional in space, in time, or both. A variety of fractional discrete data processing techniques are sketched out to model the variability of BD along with a discussion of the CTRW. The key for the learning process is the optimization method and NCW inquire into how to use the FC to improve on existing methods of optimization in ML.

A Skellam distribution is generated by taking the difference between two independent Poisson random variables, which results in an integer valued Lévy process. Gupta et al. (GKL) [8] discuss a time fractional Skellam process that describes the inter-arrival times between positive and negative jumps as a MLF distribution rather than an exponential distribution and this formulation has been applied to financial and competitive games datasets. GKL show how the formalism is extended to a Skellam process of order *k*. A Skellam process is used to model the difference between the number of goals between two teams in a soccer match. Similarly, a Skellam process of order *k* can be used to model the difference in the number of points scored by two competing teams in a basketball match where *k = 3*, meaning there are three distinct ways to score points. Elsewhere, the authors show that a fractional Skellam process is better at modeling a tick-by-tick financial dataset than the Skellam process, or equivalently that the MLF is superior to the exponential in describing the inter-arrival times between successive ticks.

The FC has the potential to improve the performance of control systems as demonstrated by Zheng et al. (ZLCW) [9]. They argue that the improvement is due to the greater flexibility in modeling of systems and in the controller design methodology using fractional-order proportional-integral-derivative (FOPID) which is a generalization of the classical PID controller. ZLCW point out that although the FOPID controller provides better performance it is also more difficult to implement. Consequently, rather than presenting a general theory they present a case study to demonstrate the advantages of the proposed method. The classical frequency-domain method is the analytic design method for the FOPID controller used in the case study.

Digital watermarking is a form of embedding a signal (a watermark) within another signal known as the cover, which might be a digital media, such as an image, audio, video, or other digital media, and has become popular as a copyright enforcement tool in the last few decades. Gonzalez-Lee et al. (GVMNPL) [10] explore the advantages of a FC watermarking system for detecting Gaussian watermarks. They briefly critique multiple FC strategies that have been adopted to replace the linear additive rule more commonly used to watermark a signal. Watermarking includes using fractional derivatives, since there is a relationship between the order of the derivative and the resulting function; fractional Fourier transforms (random, continuous, and discrete), since there is a strong dependency between the orders and the resulting coefficient set; as well as, fractional Wavelet transforms.

CVMNPL emphasize that all the techniques discussed that use the FC have the same starting point and the overall difference among them is the use of some transform coefficients set for watermarking. In a previous work, the authors investigated the case of Gaussian watermarks and their results suggested that the FC reduces the false positives percentages (FPP). In that earlier work, however, they had limited testing and a deeper study of the fractional scheme for detecting Gaussian watermarks was called for. The present work accomplishes this task and confirms that the fractional scheme is reliable for the unambiguous (error-free) detection of Gaussian watermarks.

Song and Karniadakis (SK) [11] open their contribution to the anthology with the assertion that the modeling of wall-bounded turbulent flows is presently an unsolved problem in classical physics. They propose a fundamentally new approach for modeling the entire average velocity profile from the wall to the centerline of the pipe based on the FC. They were surprised to find that representing the Reynolds stresses with a non-local variable-order fractional derivative that decays with distance from the wall results in a universal form for all Reynolds numbers for channel flow, pipe flow, and Couette flow.

A remarkable feature of this paper is the exhaustive numerical testing of the new theoretical results against existing datasets from direct numerical simulation of the equations, as well as from experimental measurements. Taken together these results support the hypothesis that the rate of turbulent diffusion changes continuously with distance from the wall and the strong non-locality of turbulent interactions intensify away from the wall.

The final paper in this anthology presents an overview of the rapidly expanding area of Distributed-Order Fractional Calculus (DOFC) by Ding et al. (DPSS) [12]. DOFC generalizes the intrinsic multiscale nature of constant-order and variable-order fractional operators, which provides new ways to think about and model systems whose behavior emerges from the complex interplay and superposition of non-local and memory effects across a multitude of scales. They discuss the various ways the fractional order in space and/or time can be distributed and review the multiple ways these equations can be numerically integrated. The areas of application on which they focus are engineering and the physical sciences, with applications to viscoelasticity, transport processes and control theory taking center stage.

Mechanisms, such as multiple relaxation time in viscoelasticity, multiple temporal and spatial effects in transport processes, and mixtures of time delays in control theory, have all illustrated the significance of DOFC over more traditional integer-order methods. This review provides a glimpse into the various ways the DOFC has established its utility in the modeling of previously unsolved or partially solved complex problems. Hopefully, the attentive reader will see a way in which the DOFC may provide insight into a problem they have put on the backburner because they could not see a way forward.
